# The multi-biomarker disease activity test for assessing response to treatment strategies using methotrexate with or without prednisone in the CAMERA-II trial

**DOI:** 10.1186/s13075-020-02293-x

**Published:** 2020-09-09

**Authors:** M. S. Jurgens, M. Safy-Khan, M. J. H. de Hair, J. W. J. Bijlsma, P. M. J. Welsing, J. Tekstra, F. P. J. G. Lafeber, E. H. Sasso, J. W. G. Jacobs, on behalf of the Society for Rheumatology research Utrecht

**Affiliations:** 1 Department of Rheumatology & Clinical Immunology, University Medical Center Utrecht, University of Utrecht, G02.228, PO Box 85500, 3508 GA Utrecht, The Netherlands; 2 Novartis Pharma BV, Amsterdam, the Netherlands; 3 Crescendo Bioscience, Inc., South San Francisco, CA USA

**Keywords:** Rheumatoid arthritis, Multi-biomarker disease activity score, DAS28, Biomarkers, CAMERA-II

## Abstract

**Objectives:**

The CAMERA-II trial compared two tight-control, treat-to-target strategies, initiating methotrexate with prednisone (MTX+pred) or MTX with placebo (MTX+plac), in early RA-patients. The multi-biomarker disease activity (MBDA) blood test objectively measures RA disease activity with a score of 1–100. In CAMERA-II, response profiles of the MBDA score, its individual biomarkers, and DAS28 were assessed.

**Methods:**

We evaluated 92 patients from CAMERA-II of whom clinical data and serum for MBDA testing at baseline and ≥ 1 time-point from months 1, 2, 3, 4, 5, 6, 9, or 12 were available. Changes (∆) from baseline for DAS28 and MBDA score and comparisons of ∆DAS28 and ∆MBDA score over time within the MTX+pred versus the MTX+plac strategy were tested for significance with *t* tests. Changes in biomarker concentration from baseline to months 1–5 were tested with Wilcoxon signed rank test and tested for difference between treatment arms by Mann-Whitney *U* test.

**Results:**

MBDA and DAS28 showed similar response profiles, with gradual improvement over the first 6 months in the MTX+plac group, and in the MTX+pred group faster improvement during month 1, followed by gradual improvement. The 12 MBDA biomarkers could be grouped into 4 categories of response profiles, with significant responses for 4 biomarkers during the MTX+plac strategy and 9 biomarkers during the MTX+pred strategy.

**Conclusions:**

MBDA tracked treatment response in CAMERA-II similarly to DAS28. More individual MBDA biomarkers tracked treatment response to MTX+pred than to MTX+plac. Four response profiles could be observed.

**Trial registration:**

CAMERA-II International Standard Randomised Controlled Trial Number: ISRCTN 70365169. Registered on 29 March 2006, retrospectively registered.

## Significance and clinical relevance

- This is the first longitudinal study of MBDA scores in MTX-based treatment strategies with or without prednisone in early RA patients.
- The MBDA score tracked treatment response in CAMERA-II, similarly to DAS28.
- The 12 MBDA biomarkers could be grouped into 4 distinct categories based on their response profile to treatment.

## Background

Rheumatoid arthritis (RA) is a chronic disease of inflammation in synovial joints, resulting in joint damage, physical disability and decreased life span. RA affects approximately 0.5–1.0% of adults in industrialized countries [1, 2]. As treatment options for RA have improved, it has become the goal of therapy to achieve remission as rapidly as possible [3–5]. Current guidelines recommend early initiation of methotrexate (MTX) as the anchor disease-modifying anti-rheumatic drug (DMARD) [6, 7]. Tight control with treat-to-target strategies, preferably including MTX [8–10], has been shown to provide better outcomes than the former standard practices [11–16]. In treat-to-target strategies, RA disease activity is quantitatively assessed at regular intervals, and based on pre-specified criteria for treatment response, treatment is adjusted to expeditiously achieve a target of low disease activity or remission [17]. Treat-to-target or tight control strategies require that physicians assess RA disease activity quantitatively. Measures based on physical examination and history, including joint counts and patient global assessment, are subjective and variable between observers. The routine inflammatory response measures of RA disease activity, erythrocyte sedimentation rate (ESR), and C-reactive protein (CRP) have the shortcoming that they are frequently in the normal range for patients with active RA and are not specific for the disease [18, 19]. Studies with magnetic resonance imaging or ultrasound have demonstrated that, even when clinically based criteria for remission are met, joint inflammation is often demonstrable and progressive damage can be ongoing [20, 21]. Thus, there is a need for objective measures that are more sensitive to joint inflammation and more accurately predict progressive joint damage than current clinical assessment tools. The multi-biomarker disease activity (MBDA) blood test measures 12 biomarkers relevant to the pathophysiology of RA to provide an objective measure of RA disease activity. It uses a validated algorithm combining the biomarker concentrations to generate an integer score on a scale of 1 to 100 [22–25]. The MBDA score correlates with the 28-joint disease activity score using CRP (DAS28-CRP) and other clinical measures of RA disease activity, and change in MBDA score correlates with change in DAS28-CRP [26]. In a study of patients with established RA receiving ongoing treatment with DMARDs, MBDA score was more strongly associated with radiographic progression than DAS28-CRP, and among patients in DAS28-CRP remission, progression was more frequent among those with a high MBDA score [27]. Similar analyses of patients from SWEFOT, a trial of tight control strategies for patients with early RA, found that baseline MBDA score was more strongly associated with radiographic progression than DAS28 or CRP [28]. Analyses of the MBDA score were mostly cross-sectional. No study yet evaluated the MBDA response longitudinally at multiple, monthly time points to MTX-based treatment strategies with or without prednisone, such as were applied in the Computer Assisted Management in Early RA Trial-II (CAMERA-II) [29]. In the present sub-study of CAMERA-II, the two strategy arms were compared longitudinally at monthly intervals to determine if the response profiles differed between the MBDA score and DAS28, or among the 12 individual biomarkers of the MBDA score.

## Methods

### CAMERA-II clinical study procedures and summary of results

The design, intervention, and main analyses of the CAMERA-II study are reported in detail elsewhere [29]. To summarize, CAMERA-II was a 2-year, prospective, randomized, placebo-controlled, double-blind multicenter tight control and treat-to-target (remission) strategy trial among patients with early RA (< 1 year since diagnosis). Patients were 18 years or older and naïve to DMARD therapy, including glucocorticoids.

At study baseline, all patients initiated a monthly step-up strategy using oral MTX, at a starting dosage of 10 mg per week, and were randomized to also receive either oral prednisone, 10 mg per day (MTX+pred strategy), or placebo (MTX+plac strategy). Rheumatologists assessed each patient monthly, and a computer program indicated whether the patient had achieved response (> 20% improvement) compared with the previous visit. If response was not sufficient and remission had not been achieved, MTX dosage was increased by 5 mg per week until the patient had achieved remission (swollen joint count (SJC) = 0 and ≥ 2 of the following criteria: tender joint count (TJC) ≤ 3, visual analogue scale (VAS) score ≤ 20 mm, and ESR ≤ 20 mm/h). At the maximum (30 mg per week) or maximum tolerable MTX dosage, if a step-up in treatment was indicated, MTX was administered at the same dosage subcutaneously. As the next step, cyclosporine was added to the regimen. However, shortly after start of the trial, cyclosporine was replaced with adalimumab [29]. All patients received folic acid, calcium carbonate with vitamin D and a bisphosphonate. The medical ethics committee of the University Medical Center Utrecht had approved the study. All patients had provided written informed consent before entering the study.

Onset of efficacy was more rapid in the MTX+pred strategy group, and at 2 years, the MTX+pred strategy group had achieved a greater reduction in disease activity, as measured with the DAS28, and had less progression of erosive joint damage, fewer adverse effects, and less frequent need for additional biological (b) DMARD treatment [18].

### The MBDA score

The development and validation of the MBDA score are reported in detail elsewhere [24, 25]. In short, 130 candidate biomarkers were tested in feasibility studies, of which 12 were selected for final algorithm development and validation. The biomarker selection and algorithm were optimized to maximize the strength of the association of the MBDA score with DAS28-CRP in a cohort of patients on diverse treatments [25]. Concentrations of these 12 MBDA protein biomarkers (CRP, epidermal growth factor, interleukin (IL) 6, leptin, matrix metalloproteinase 1 (MMP-1), matrix metalloproteinase 3 (MMP-3), resistin, serum amyloid A (SAA), tumor necrosis factor receptor type I (TNF-RI), vascular cell adhesion molecule 1 (VCAM-1), vascular endothelial growth factor A (VEGF-A), and cartilage glycoprotein 39 (YKL-40)) were measured by multiplex immunoassay using the Meso Scale Discovery MULTI-ARRAY® platform. Biomarker concentrations were combined in the validated MBDA algorithm to generate the MBDA score, an integer from 1 to 100, for which the established categories of disease activity are low (< 30), moderate (30–44), and high (> 44) [24]. Biomarker measurement and MBDA score calculation were performed in the CLIA-certified laboratory of Crescendo Bioscience, Inc., South San Francisco, CA, USA, using the same instrument, reagents, and algorithm as for the Vectra® DA test, which is commercially available in the USA.

### Multiple biomarker-based disease activity assessment in CAMERA-II

MBDA biomarkers were evaluated in serum samples obtained at baseline and at 1, 2, 3, 4, 5, 6, 9, and 12 months. Numbers of samples available for the present study varied between time points, based on patient compliance and the volume of available sample. Of 104 patients in CAMERA-II for whom baseline sera were available for MBDA testing, MBDA scores and DAS28 were analyzed for the 92 who had at least one MBDA test result for months 1, 2, 3, 4, 5, 6, 9, or 12. For this 92-patient cohort, the average number of post-baseline tests per patient was 3.7.

### Statistical analyses

To evaluate changes from baseline for DAS28 and MBDA score and comparisons of change in DAS28 or MBDA score over time between patients treated with the MTX+pred or MTX+plac strategy, a *t* test was performed for each time point evaluated. Association between change from baseline to 12 months for DAS28 and MBDA score was assessed using Spearman’s rank correlation.

Concentrations of individual biomarkers were analyzed for the subset of 51 patients who had an MBDA test at baseline and at least one time point from months 1 to 5, to focus on the initial biomarker responses to treatment and exclude possible effects from exposure to cyclosporine or adalimumab. The average number of post-baseline tests per patient was 3.3 in this subset. Biomarker concentrations were analyzed after base-10 logarithm (log10) transformation, to approximate a normal distribution. The changes from baseline in log10 biomarker concentrations were assessed for months 1–5 for each treatment arm by Wilcoxon signed rank test and compared between treatment arms by Mann-Whitney *U* tests. The means of the changes were calculated as averages of individual changes in log10 values, and standard error (SE) values were determined accordingly. For presentation in graphs, each mean change (D) was back-transformed by raising 10 to the *D* power, thus reversing the log10 transformation to generate a fractional value, relative to baseline, on a linear scale. Thus, any time point demonstrating no change from baseline was represented on the graph with a value of 1.0, and for example, a 20% reduction from baseline was represented with a value of 0.8. Response profiles are the courses of changes from baseline for the MTX+plac and MTX+pred strategy arms.

For the individual biomarkers, profile categories were defined, dependent on their response to MTX+plac, and their response to concomitant prednisone, i.e., the difference in response to MTX+plac and MTX+pred. This was based on visual inspection of curves representing change from baseline in biomarker concentration for each treatment strategy arm and on *p* values for changes from baseline and for the difference between treatment strategy arms. The software package R 2.15.1 (www.r-project.org) was used for the analyses. No clinical or biomarker data were imputed. A *p* value of < 0.05 was considered statistically significant. No adjustments were made for multiple testing.

## Results

### Baseline characteristics

Baseline characteristics of the 92 patients analyzed to month 12 were similar between treatment arms (Table 1). Characteristics were similar between these 92 patients and the subset of 51 patients for whom individual biomarkers were analyzed to month 5 (data not shown), and the 236 patients of the full CAMERA-II population, except for joint counts and CRP, which tended to be lower in the present study.

**Table 1:** Patients’ characteristics at baseline

	All patients ***N*** = 92	MTX+plac ***n*** = 50	MTX+pred ***n*** = 42
% female	59	56	62
Age	57 (47–65)	54 (46–65)	58 (47–67)
Smoking, as number of cigarettes per day	0 (0–5)	0 (0–0.5)	0 (0–5)
RF status, % positive	63	70	56
HAQ score, 0–3	1.1 (0.63–1.6)	1.2 (0.63–1.6)	1.1 (0.63–1.5)
General health VAS, 0–10	5.0 (2.7–6.7)	5.1 (3.6–6.6)	4.7 (2.2–6.7)
TJC28	10 (6–17)	9.5 (6–13)	12 (5–18)
SJC28	11 (7–15)	11 (7–15)	11 (6–15)
ESR mm/h	31 (19–44)	29 (19–43)	31 (18–45)
CRP mg/L	16 (2.7–41)	16 (5.5–42)	16 (1.9–37)
DAS28	5.6 (4.9–6.6)	5.6 (5–6.3)	5.6 (4.1–6.9)
MBDA score, 1–100	51 (39–71)	54 (40–72)	49 (40–70)

No statistically significant differences in baseline characteristic between MTX+plac and MTX+pred groups. Values are median (interquartile range) or percentage

*RF*, rheumatoid factor (RF status was available for 82 of 92, 43 of 50, and 39 of 42 patients, respectively); *MTX*, methotrexate; *HAQ*, health assessment questionnaire; *VAS*, visual analogue scale general health; *TJC28*, tender joint count assessing 28 joints; *SJC28*, swollen joint count assessing 28 joints; *ESR*, erythrocyte sedimentation rate; *CRP*, C-reactive protein; *DAS28*, 28-joint-based disease activity score; *MBDA*, multi-biomarker disease activity; higher scores of HAQ, VAS, and MBDA score reflect worse scores; *MTX+plac*, the methotrexate and placebo strategy; *MTX+pred* the methotrexate and prednisone strategy

### Clinical, MBDA, and biomarker responses to therapy

**Fig. 1:**
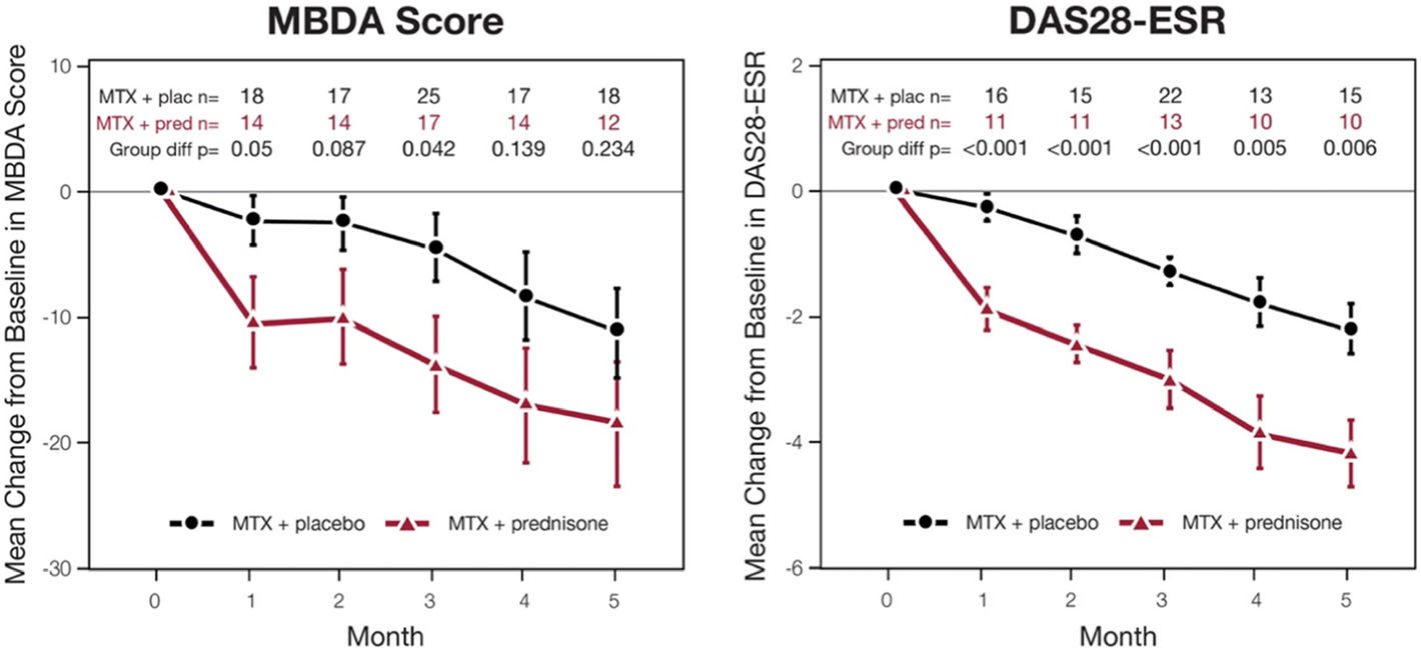
Mean (SE) changes from baseline in MBDA score and DAS28 for each strategy arm of the CAMERA-II study. Mean (SE) changes from baseline in MBDA score and DAS28 for each strategy arm of the CAMERA-II study are shown over first 5 months at monthly assessments, each prior to dosing with MTX and placebo (MTX+plac) or MTX and prednisone (MTX+pred) at that time-point. *P* values of *t* tests for comparison between strategy arms (Group diff). Patient numbers are shown for each time-point in each strategy arm

Reductions in DAS28 and MBDA score had similar profiles of change from baseline over time, with more rapid and greater initial responses observed for patients treated with the MTX+pred strategy, compared with the MTX+plac strategy (Table 2, Fig. 1). For the 59 patients with data at baseline and 12 months, the changes from baseline to 12 months for DAS28 and MBDA score were significantly correlated, both overall (*r* = 0.56, *p* < 0.001) and within each treatment arm: MTX+pred (*n* = 28, *r* = 0.57, *p* = 0.002); MTX+plac (*n* = 31, *r* = 0.57, *p* = 0.001).

**Table 2:** Mean changes from baseline for DAS28 and MBDA score during treatment with a tight-control strategy initiating MTX+plac or MTX+pred

	DAS28				MBDA score			
	MTX+plac		MTX+pred		MTX+plac		MTX+pred	
Time-point (month)	***n***	Mean change	***N***	Mean change	***n***	Mean change	***n***	Mean change
1	16	− 0.3 *P* = 0.243	11	− 1.9 *P* < 0.001	18	− 3 *P* = 0.265	14	− 12 *P* = 0.013
2	15	− 0.7 *P* = 0.021	11	− 2.4 *P* < 0.001	17	− 3 *P* = 0.251	14	− 11 *P* = 0.02
3	22	− 1.3 *P* < 0.001	13	− 3.0 *P* < 0.001	25	− 5 *P* = 0.093	17	− 15 *P* = 0.002
4	13	− 1.8 *P* = 0.001	10	− 3.9 *P* < 0.001	17	− 9 *P* = 0.029	14	− 19 *P* = 0.003
5	15	− 2.2 *P* < 0.001	10	− 4.2 *P* < 0.001	18	− 12 *P* = 0.006	12	− 20 *P* = 0.003
6	18	− 2.8 *P* < 0.001	12	− 3.0 *P* = 0.001	29	− 20 *P* < 0.001	19	− 16 *P* = 0.001
9	17	− 2.7 *P* < 0.001	12	− 3.2 *P* = 0.001	24	− 24 *P* < 0.001	17	− 20 *P* = 0.001
12	31	− 2.8 *P* < 0.001	28	− 3.1 *P* < 0.001	44	− 20 *P* < 0.001	37	− 16 *P* < 0.001

Each *n* value indicates number of patients from the study cohort (total *N* = 92) with available data at that time-point. *P* values are for changes from baseline by *t* test

*DAS28* 28-joint-based disease activity score, *MBDA* multi-biomarker disease activity, *MTX+plac* the methotrexate and placebo strategy, *MTX+pred* the methotrexate and prednisone strategy

**Fig. 2:**
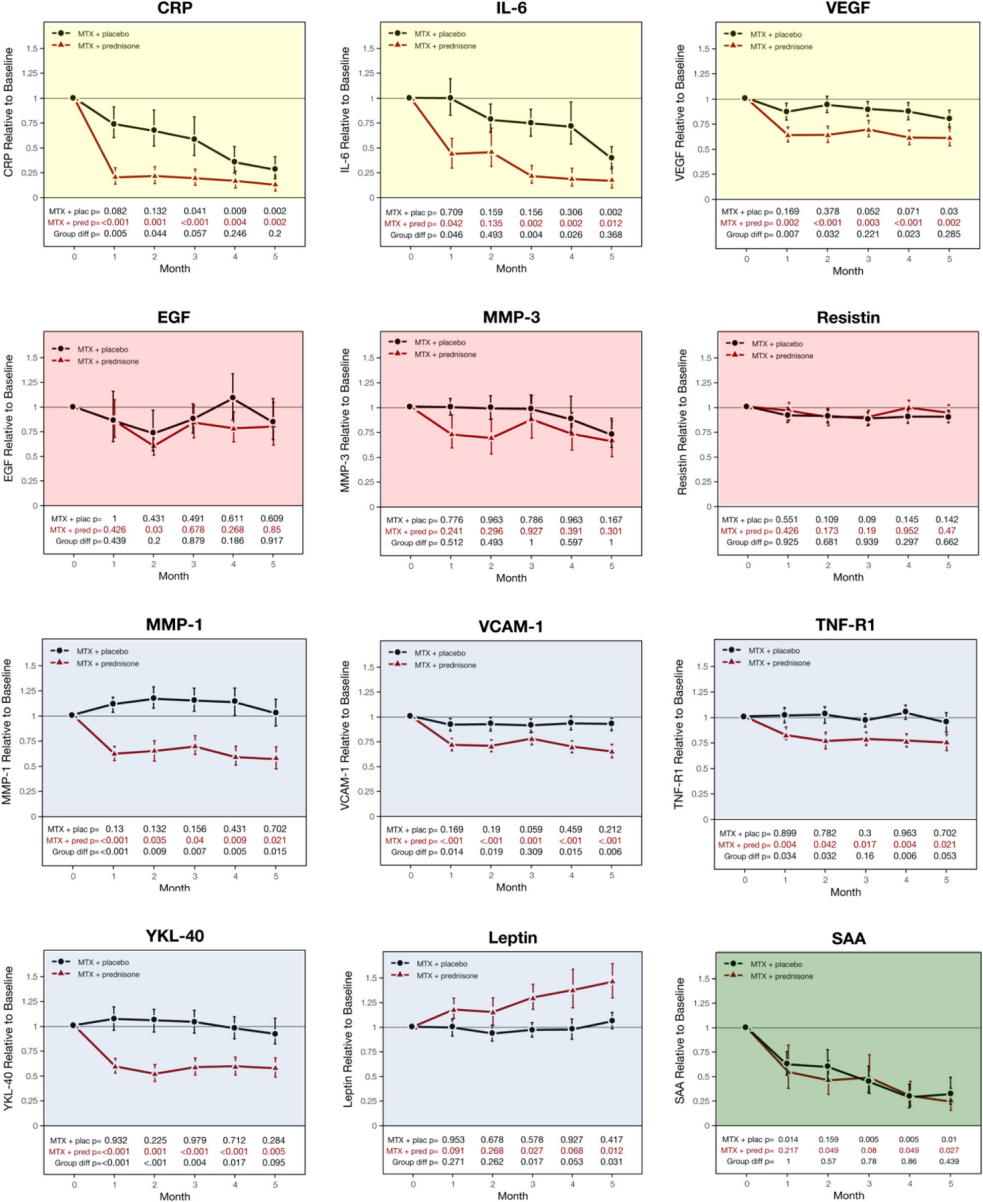
Mean (SE) changes in concentrations of MBDA biomarkers over the first 5 months of the CAMERA-II study. Mean (SE) changes in concentrations of MBDA biomarkers over the first 5 months of the CAMERA-II study Each measurement was performed in serum obtained prior to dosing with MTX and placebo (MTX+plac) or MTX and prednisone (MTX+pred) at that time-point. Means and standard errors were calculated using log10-transformed values of the biomarker concentrations and, for graphic display, were then converted to fractional values relative to baseline on a linear scale (see the “Methods” section). Patient numbers for each time-point in each strategy arm are the same as in the MBDA panel of Fig. 1. Four profiles of biomarker response categories can be observed: (1) statistically significant gradual reduction in biomarker concentration with the MTX+plac strategy versus a more rapid and statistically significantly greater reduction in biomarker concentration with the MTX+pred strategy (CRP, IL-6, VEGF), yellow background in figure; (2) little (but not statistically significant, except for EGF only at month 2 in MTX+pred), or no response to either treatment, with no significant difference between strategies (EGF, MMP-3, resistin), red background in figure; (3) little (but not statistically significant) or no biomarker response with the MTX+plac strategy versus a more rapid and statistically significantly greater biomarker response with the MTX+pred strategy (decrease: MMP-1, VCAM-1, TNF-R1, YKL-40; increase: leptin), blue background in figure; and (4) significant gradual reduction in biomarker concentration over time with the MTX+plac strategy and the MTX+pred strategy, with no statistically significant difference between the two arms (SAA), green background in figure

Most individual MBDA biomarkers showed statistically significant changes over time, and for eight biomarkers, these changes differed between treatment arms (Fig. 2). Treatment with MTX+plac induced a statistically significant decline in concentration for 4 of the biomarkers: CRP, IL-6, SAA, and VEGF. Treatment with MTX+pred significantly decreased the concentrations of these 4 biomarkers and also MMP-1, TNF-R1, VCAM-1, and YKL-40. No sustained, significant decrease was observed with either treatment for MMP-3, EGF, or resistin. Leptin concentrations were unaffected by treatment with MTX+plac, but they increased with MTX+pred. When considering the two treatment arms in tandem, 4 profiles of biomarker response categories can be observed (Fig. 2).

## Discussion

The present study is the first to present the MBDA score at multiple, consecutive monthly time points following initiation of MTX-based treatment strategies, with or without prednisone. This frequency of testing allowed us to demonstrate that the added benefit from prednisone was almost entirely achieved within the first month, both clinically and in terms of biomarker measurements. Since prednisone provides symptomatic benefit rapidly after the first dose, physiological effects of prednisone in CAMERA-II probably started before the first post-baseline assessment at 1 month. The response of CRP, which is a component of the MBDA score, can precede clinical response with biologics [30, 31]. We found that the MBDA score declined steadily over the first 6 months of treatment with MTX+plac. For patients who received MTX+pred, the initial MBDA response was markedly greater than for those in the MTX+plac arm and was followed by a gradual, continued decline in MBDA score that approximately paralleled that of the MTX+plac arm. This profile of MBDA response resembled the clinical response, as assessed with DAS28. These findings are consistent with the fact that the MBDA score was validated based on its correlation with DAS28-CRP, DAS28, and other clinically based measures of disease activity [24, 25]. Similarly, change in MBDA score correlated here with change in DAS28 in both treatment arms, which is consistent with previous analyses of changes in MBDA score, DAS28-CRP, and DAS28 [25, 26]. For both DAS28 and MBDA score, responses during the second 6 months of treatment were similar between treatment arms and relatively stable, consistent with overall results of the study [18]. While the response profiles for MBDA score and DAS28 appeared to be similar in this study, the changes from baseline and the differences between treatment arms were statistically significant at more time points for DAS28 than for the MBDA score. In addition, the DAS28 responses had narrower confidence intervals (Fig. 2). These differences between MBDA score and DAS28 may be reflections of the tight control strategy in CAMERA-II, where treatment adjustments were based on the DAS28 component measures. An opposite result might have been obtained if, instead, the MBDA score had been used for dictating the tight-control strategy. Such results are not currently available. The similarity of the CAMERA-II response profiles for DAS28 and MBDA score, which are both composite measures, led us to examine whether the individual biomarkers of the MBDA test also exhibited similar patterns of change over time. We found that, while some biomarkers had similar response profiles to that of the MBDA score, considerable variability was observed among the 12 biomarkers. Leptin concentrations were unaffected by treatment with MTX+plac, but unlike any of the other biomarkers, they increased with MTX+pred. This result is consistent with findings in patients treated with glucocorticoids alone [32, 33]. By contrast, leptin concentrations have been reported to not change significantly from baseline during treatment with an anti-TNF agent and concomitant MTX, with or without a concomitant glucocorticoid [34, 35]. For SAA, a significant response was seen with MTX alone, but prednisone provided no additional effect. This category profile was unique to SAA and contrasts with that of CRP and IL-6, even though CRP and SAA are both acute phase proteins of which the production is driven by IL-6 [36]. The basis for this lack of prednisone effect on SAA is uncertain. Evidence that SAA is a more sensitive indicator of inflammation than CRP [37], and that glucocorticoids can increase the production of SAA outside of the liver [38, 39], suggests that the basis for our SAA finding may be multifactorial. The most conclusive findings in this study came from comparing the biomarkers in terms of their response profiles, i.e., the patterns observed by viewing the two treatment arms in tandem for each biomarker. The 12 MBDA biomarkers could be grouped into 4 categories based on their response profile to the MTX+pred and MTX+plac strategies. Whether or not a biomarker responded to MTX+plac, a greater response was usually observed with the addition of prednisone, as seen with CRP, IL-6, and VEGF, which decreased in both arms but to a greater degree in the MTX+pred arm. Another category profile was seen with MMP-1, VCAM-1, TNF-R1, YKL-40, and leptin, which did not seem to respond to MTX+plac, but did respond to MTX+pred, thus to prednisone. These two profiles suggest that prednisone affects a broader spectrum of immunosuppressive mechanisms than MTX. A limitation of this study is that it was a post hoc analysis of 92 of the 236 patients of CAMERA-II. Although the patients studied here were selected on the basis of availability of serum samples, their baseline data were similar between randomization arms and, overall, to those of the full CAMERA-II population. Moreover, although sample size at individual time-points was small and it varied across time-points, statistical significance was achieved for all single-biomarker values that were interpreted as being changed from baseline or as being different between the two arms. Subset analyses were not performed, due to the limited sample size. Given that patient numbers were identical for the 12 biomarkers, the distinctiveness of the 4 categories of biomarker response profile suggests that they reflect true biological differences. The results obtained here are hypothesis-generating and suggest that a larger study is warranted for confirmation and further exploration.

## Conclusions

In summary, during the first year of the CAMERA-II trial, the MBDA score and DAS28 were similar in their detection of response to treatment strategies initiating MTX with placebo or MTX and prednisone. Like the DAS28, the MBDA score demonstrated a more rapid and greater early response to MTX with prednisone compared with MTX with placebo. Analysis of the 12 MBDA biomarkers showed that more biomarkers responded to MTX with prednisone than to MTX with placebo, with 4 distinct categories of response profile observed.

## Data Availability

The data that support the findings of this study are available from [FPJGL] but restrictions apply to the availability of these data, which were used under license for the current study, and so are not publicly available. Data are however available from the authors upon reasonable request and with permission of [FPJGL].

## Abbreviations

CAMERA-II: Computer Assisted Management in Early Rheumatoid Arthritis Trial-II
MTX: Methotrexate
pred: Prednisone
plac: Placebo
RA: Rheumatoid arthritis
DMARD: Disease-modifying anti-rheumatic drug
ESR: Erythrocyte sedimentation rate
CRP: C-reactive protein
MBDA: Multi-biomarker disease activity
DAS29-CRP: 28-joint disease activity score using CRP
SJC: Swollen joint count
TJC: Tender joint count
VAS: Visual analogue scale
bDMARD: Biological disease-modifying anti-rheumatic drug
EPG: Epidermal growth factor
IL-6: Interleukin 6
MMP-1: Matrix metalloproteinase 1
MMP-3: Matrix metalloproteinase 3
SAA: Serum amyloid A
TNF-RI: Tumor necrosis factor receptor type I
VCAM-1: Vascular cell adhesion molecule 1
VEGF-A: Vascular endothelial growth factor A
YKL-40: Cartilage glycoprotein 39
SE: Standard error
D: Mean change

## References

[1] Scott DL, Wolfe F, Huizinga TW (2010). Rheumatoid arthritis. Lancet..

[2] Mitchell DM, Spitz PW, Young DY, Bloch DA, McShane DJ, Fries JF (1986). Survival, prognosis, and causes of death in rheumatoid arthritis. Arthritis Rheum.

[3] Resman-Targoff BH, Cicero MP (2010). Aggressive treatment of early rheumatoid arthritis: recognizing the window of opportunity and treating to target goals. Am J Manag Care.

[4] van Hulst LT, Hulsch ME, van Riel PL (2011). Achieving tight control in rheumatoid arthritis. Rheumatology..

[5] Wilske KR, Healey LA (1989). Remodeling the pyramid--a concept whose time has come. J Rheumatol.

[6] Bakker MF, Jacobs JW, Welsing PM, van der Werf JH, Linn-Rasker SP, van der Veen MJ (2010). Are switches from oral to subcutaneous methotrexate or addition of ciclosporin to methotrexate useful steps in a tight control treatment strategy for rheumatoid arthritis? A post hoc analysis of the CAMERA study. Ann Rheum Dis.

[7] Sokka T, Envalds M, Pincus T (2008). Treatment of rheumatoid arthritis: a global perspective on the use of antirheumatic drugs. Mod Rheumatol.

[8] Choy EH, Smith C, Dore CJ, Scott DL (2005). A meta-analysis of the efficacy and toxicity of combining disease-modifying anti-rheumatic drugs in rheumatoid arthritis based on patient withdrawal. Rheumatology..

[9] Pincus T, Yazici Y, Sokka T, Aletaha D, Smolen JS (2003). Methotrexate as the “anchor drug” for the treatment of early rheumatoid arthritis. Clin Exp Rheumatol.

[10] Jacobs JW. Optimal use of non-biologic therapy in the treatment of rheumatoid arthritis. Rheumatology. 2012;51(Suppl 4):iv3–8. 10.1093/rheumatology/kes083 22513146

[11] Goekoop-Ruiterman YPM, De Vries-Bouwstra JK, Kerstens PJSM, Nielen MM, Vos K, van Schaardenburg D (2010). DAS-driven therapy versus routine care in patients with recent-onset active rheumatoid arthritis. Ann Rheum Dis.

[12] Verstappen SM, Jacobs JW, van der Veen MJ, Heurkens AH, Schenk Y, ter Borg EJ (2007). Intensive treatment with methotrexate in early rheumatoid arthritis: aiming for remission. Computer Assisted Management in Early Rheumatoid Arthritis (CAMERA, an open-label strategy trial). Ann Rheum Dis.

[13] Jurgens MS, Welsing PM, Jacobs JW (2012). Overview and analysis of treat-to-target trials in rheumatoid arthritis reporting on remission. Clin Exp Rheumatol.

[14] Grigor C, Capell H, Stirling A, McMahon AD, Lock P, Vallance R (2004). Effect of a treatment strategy of tight control for rheumatoid arthritis (the TICORA study): a single-blind randomised controlled trial. Lancet..

[15] Schipper LG, van Hulst LT, Grol R, van Riel PL, Hulscher ME, Fransen J (2010). Meta-analysis of tight control strategies in rheumatoid arthritis: protocolized treatment has additional value with respect to the clinical outcome. Rheumatology..

[16] Schipper LG, Vermeer M, Kuper HH, Hoekstra MO, Haagsma CJ, Den Broeder AA (2011). A tight control treatment strategy aiming for remission in early rheumatoid arthritis is more effective than usual care treatment in daily clinical practice: a study of two cohorts in the Dutch Rheumatoid Arthritis Monitoring registry. Ann Rheum Dis.

[17] Bakker MF, Jacobs JW, Verstappen SM, Bijlsma JW (2007). Tight control in the treatment of rheumatoid arthritis: efficacy and feasibility. Ann Rheum Dis.

[18] Sokka T, Pincus T (2009). Erythrocyte sedimentation rate, C-reactive protein, or rheumatoid factor are normal at presentation in 35%-45% of patients with rheumatoid arthritis seen between 1980 and 2004: analyses from Finland and the United States. J Rheumatol.

[19] Kay J, Morgacheva O, Messing SP, Kremer JM, Greenberg JD, Reed GW (2014). Clinical disease activity and acute phase reactant levels are discordant among patients with active rheumatoid arthritis: acute phase reactant levels contribute separately to predicting outcome at one year. Arthritis Res Ther.

[20] Lillegraven S, Prince FH, Shadick NA, Bykerk VP, Lu B, Frits ML (2012). Remission and radiographic outcome in rheumatoid arthritis: application of the 2011 ACR/EULAR remission criteria in an observational cohort. Ann Rheum Dis.

[21] Brown AK, Conaghan PG, Karim Z, Quinn MA, Ikeda K, Peterfy CG (2008). An explanation for the apparent dissociation between clinical remission and continued structural deterioration in rheumatoid arthritis. Arthritis Rheum.

[22] Bakker MF, Cavet G, Jacobs JW, Bijlsma JW, Haney DJ, Shen Y (2012). Performance of a multi-biomarker score measuring rheumatoid arthritis disease activity in the CAMERA tight control study. Ann Rheum Dis.

[23] Eastman PS, Manning WC, Qureshi F, Haney D, Cavet G, Alexander C (2012). Characterization of a multiplex, 12-biomarker test for rheumatoid arthritis. J Pharm Biomed Anal.

[24] Curtis JR, van der Helm-van Mil AH, Knevel R, Huizinga TW, Haney DJ, Shen Y (2012). Validation of a novel multi-biomarker test to assess rheumatoid arthritis disease activity. Arthritis Care Res.

[25] Centola M, Cavet G, Shen Y, Ramanujan S, Knowlton N, Swan KA (2013). Development of a multi-biomarker disease activity test for rheumatoid arthritis. PLoS One.

[26] Hirata S, Dirven L, Shen Y, Centola M, Cavet G, Lems WF (2013). A multi-biomarker score measures rheumatoid arthritis disease activity in the BeSt study. Rheumatology..

[27] van der Helm-van Mil AH, Knevel R, Cavet G, Huizinga TW, Haney DJ (2013). An evaluation of molecular and clinical remission in rheumatoid arthritis by assessing radiographic progression. Rheumatology..

[28] Hambardzumyan K, Bolce R, Saevarsdottir S, Cruickshank SE, Sasso EH, Chernoff D (2015). Pretreatment multi-biomarker disease activity score and radiographic progression in early RA: results from the SWEFOT trial. Ann Rheum Dis.

[29] Bakker MF, Jacobs JW, Welsing PM, Verstappen SM, Tekstra J, Ton E (2012). Low-dose prednisone inclusion in a methotrexate-based, tight control strategy for early rheumatoid arthritis. A Randomized Trial. Ann Intern Med.

[30] Schiff M, Weinblatt ME, Valente R, van der Heijde D, Citera G, Elegbe A (2013). Head-to-head comparison of subcutaneous abatacept versus adalimumab for rheumatoid arthritis: two-year efficacy and safety findings from AMPLE trial. Ann Rheum Dis.

[31] Genovese MC, McKay JD, Nasonov EL, Mysler EF, da Silva NA, Alecock E (2008). Interleukin-6 receptor inhibition with tocilizumab reduces disease activity in rheumatoid arthritis with inadequate response to disease-modifying antirheumatic drugs: the tocilizumab in combination with traditional disease-modifying antirheumatic drug therapy study. Arthritis Rheum.

[32] Cimmino MA, Andraghetti G, Briatore L, Salani B, Parodi M, Cutolo M (2010). Changes in adiponectin and leptin concentrations during glucocorticoid treatment: a pilot study in patients with polymyalgia rheumatica. Ann N Y Acad Sci.

[33] Klaasen R, Herenius MM, Wijbrandts CA, de Jager W, van Tuyl LH, Nurmohamed MT (2012). Treatment-specific changes in circulating adipocytokines: a comparison between tumour necrosis factor blockade and glucocorticoid treatment for rheumatoid arthritis. Ann Rheum Dis.

[34] Harle P, Sarzi-Puttini P, Cutolo M, Straub RH (2006). No change of serum levels of leptin and adiponectin during anti-tumour necrosis factor antibody treatment with adalimumab in patients with rheumatoid arthritis. Ann Rheum Dis.

[35] Ferraz-Amaro I, Arce-Franco M, Muniz J, López-Fernández J, Hernández-Hernández V, Franco A (2011). Systemic blockade of TNF-alpha does not improve insulin resistance in humans. Horm Metab Res.

[36] Ganapathi MK, May LT, Schultz D, Brabenec A, Weinstein J, Sehgal PB (1988). Role of interleukin-6 in regulating synthesis of C-reactive protein and serum amyloid a in human hepatoma cell lines. Biochem Biophys Res Commun.

[37] Smith JW, Colombo JL, McDonald TL (1992). Comparison of serum amyloid a and C-reactive protein as indicators of lung inflammation in corticosteroid treated and non-corticosteroid treated cystic fibrosis patients. J Clin Lab Anal.

[38] Viguerie N, Picard F, Hul G, Roussel B, Barbe P, Iacovoni JS (2012). Multiple effects of a short-term dexamethasone treatment in human skeletal muscle and adipose tissue. Physiol Genomics.

[39] Smith JW, McDonald TL (1992). Production of serum amyloid A and C-reactive protein by HepG2 cells stimulated with combinations of cytokines or monocyte conditioned media: the effects of prednisolone. Clin Exp Immunol.

